# Assessing Agreement in Exposure Classification between Proximity-Based Metrics and Air Monitoring Data in Epidemiology Studies of Unconventional Resource Development

**DOI:** 10.3390/ijerph16173055

**Published:** 2019-08-23

**Authors:** Judy Wendt Hess, Gerald Bachler, Fayaz Momin, Krystal Sexton

**Affiliations:** 1 Shell Health Risk Science Team, Shell Oil Company, 150 North Dairy Ashford, Houston, TX 77079, USA; 2 Shell Health Risk Science Team, Shell International B.V., Carel Van Bylandtlaan 16, 2596 HR The Hague, The Netherlands

**Keywords:** hydraulic fracturing, unconventional development, exposure measure

## Abstract

Recent studies of unconventional resource development (URD) and adverse health effects have been limited by distance-based exposure surrogates. Our study compared exposure classifications between air pollutant concentrations and “well activity” (WA) metrics, which are distance-based exposure proxies used in Marcellus-area studies to reflect variation in time and space of residential URD activity. We compiled Pennsylvania air monitoring data for benzene, carbon monoxide, nitrogen dioxide, ozone, fine particulates and sulfur dioxide, and combined this with data on nearly 9000 Pennsylvania wells. We replicated WA calculations using geo-coordinates of monitors to represent residences and compared exposure categories from air measurements and WA at the site of each monitor. There was little agreement between the two methods for the pollutants included in the analysis, with most weighted kappa coefficients between −0.1 and 0.1. The exposure categories agreed for about 25% of the observations and assigned inverse categories 16%–29% of the time, depending on the pollutant. Our results indicate that WA measures did not adequately distinguish categories of air pollutant exposures and employing them in epidemiology studies can result in misclassification of exposure. This underscores the need for more robust exposure assessment in future analyses and cautious interpretation of these existing studies.

## 1. Introduction

Advances in onshore oil and gas development have occurred rapidly in the past decade, driven largely by unconventional resource development (URD), and specifically, hydraulic fracturing (or ‘fracking’) of horizontal wells [1]. This technology has enabled the extraction of oil and gas from shale formations and has led to the U.S. becoming the world’s top producer of oil and natural gas [2]. The largest shale formation in North America is the Marcellus, underlying nearly 90,000 square miles in portions of Pennsylvania, West Virginia, Ohio, Maryland and New York, including 60% of land area in the state of Pennsylvania. It is also one of the most prolific fields in the world, producing nearly 6 trillion cubic feet of natural gas in 2018 [3].

Unconventional well development occurs in four phases [4]. First, “pad preparation” involves clearing and leveling the proposed well site and creating access roads to the site for machinery and workers. The “drilling” phase then begins with initial drilling into the ground and concludes when the hole (i.e., wellbore) is completed and cement casing is applied. The third phase, “fracturing”, (a.k.a., hydraulic fracturing, fracking, or stimulation) involves pumping fracturing fluids into the wellbore under high pressure to enable the release of gas and/or oil held in the rock formation. Components of these fluids are transported by truck to the well site throughout the fracturing phase. Finally, “production” describes the phase in which hydrocarbons flow towards the surface through the well. While the phases are sequential, there can be gaps of days to years between them, resulting in periods of intense activity followed by little or no activity at the well. The phases differ both in duration and in their potential for air quality impacts, an important consideration when characterizing potential community exposures near well sites [4].

While conventional oil and gas development has occurred in the Marcellus for nearly 150 years, the introduction of unconventional development to the region in 2008 led to a dramatic increase in onshore oil and gas activity and a greater dispersion of wells near residential areas; with this rapid growth have come concerns of potential air quality and community health impacts [4]. Recent reports have assessed URD-related air emissions in Pennsylvania. A 2015 Pennsylvania Department of Environmental Protection (DEP) inventory of point source emissions from unconventional natural gas operations (including well sites and compressor stations) reported year-on-year increases in methane and volatile organic compounds (VOCs) but decreased emissions of nitrogen oxides (NO_x_), sulfur oxides (SO_x_) and fine particulate matter (PM_2.5_), noting that the changes occurred during a period of significant increase in production [5]. Based on the 2015 inventory, the percent of statewide stationary source emissions of NO_x_, PM_2.5_, SO_x_ and VOCs attributable to URD was 12%, 7%, 0.1% and 23%, respectively [6].

Other studies have assessed potential URD-related air quality impacts. Maskrey et al. collected continuous samples of total VOCs over a three-month period, as well as intermittent 24-h samples of 62 individual VOCs, at a school and residence that were within 900 m of a well pad [7]. VOC’s measured both during well development (including fracturing and flaring) and while the well was inactive were similar to each other, and to background concentrations in an area without drilling operations [7]. A mobile monitoring campaign in northeastern and southwestern Pennsylvania reported increased methane concentrations between 2012 and 2015, but concentrations were not correlated with either well density or average production rates at the locations sampled [8]. Methane, ethane and carbon monoxide (CO) levels in the rural Marcellus areas resembled urban concentrations, indicating a possible effect of these operations on local air quality, however other aromatic VOC emissions from URD did not appear to significantly impact local background air concentrations [8]. The DEP completed a long-term monitoring project comparing shale area and background sampling sites, including measurements of ozone (O_3_), NO_x_, CO, PM_2.5_ and other hazardous pollutants, and found little evidence of air quality impacts from unconventional gas operations [9]. The DEP also collected short-term air pollutant samples in three regions of the state in 2010 and concluded that the measured concentrations did not reach the levels expected to cause acute health impacts. Natural gas constituents were detected near drilling operations, and some compounds exceeded odor thresholds, however, no samples of CO, nitrogen dioxide (NO_2_), sulfur dioxide (SO_2_) or O_3_ exceeded the National Ambient Air Quality Standards [10].

While URD-related hazards, including criteria pollutants and VOCs, have been identified, evidence demonstrating a pathway of exposure to communities at levels sufficient to increase the risk of adverse health effects, remains lacking. The body of literature on potential health impacts of URD is increasing, however, limitations in study methodology, particularly in the area of exposure assessment, have made it difficult to establish clear links between reported health effects and URD exposure [11]. A series of Marcellus-area epidemiology studies reported statistically significant associations between URD exposure intensity and asthma, low birthweight, migraines and sinus symptoms. In these studies, exposure was based on calculations of phase-specific “well activity” (WA), representing the intensity of URD operations near a residence [12,13,14]. While these WA exposure metrics are fundamentally a function of the distance between wells and homes, they also incorporate differences in duration and potential for air quality impacts across phases of development. However, they differ from other proximity-based exposure surrogates in that they include all wells (statewide) in the exposure calculation of a study subject, with no buffer distance applied around either the residence or emission source. These defined buffer areas are typically used so that estimated pollutant exposure can be reasonably attributed to the emission source under study, and not to other point or mobile sources of the same pollutant [15,16]. The WA methodology is based on the premise (with respect to exposure via the air pathway) that residents with more and/or closer wells (i.e., higher well density) have higher exposure to URD-related pollutants, and assume that (1) all wells in the state continuously emit air pollutants, (2) pollutant emissions are the same for every well in a specific phase of production (i.e., pad preparation, drilling, fracturing, production), (3) every well in the state contributes to the URD-related exposure of every resident of the state, with the well’s contribution toward total exposure primarily dependent on distance to the residence, and (4) a subject’s exposure is based solely on well emissions and not influenced by emissions from other non-URD point and mobile sources or wind direction.

The results of these epidemiology studies have been frequently cited in calls to limit or ban URD in the US and Europe [17,18]. To our knowledge, however, there has been no assessment of whether categorizing URD exposure using these WA calculations is a valid approach, or alternatively, whether it has the potential to introduce significant exposure measurement error. The aim of our study was to evaluate whether these metrics differentiate, with some level of accuracy, levels of exposure to air pollutants that have been associated with URD activity. WA metrics can be calculated in relation to any geocoded location, whether a residence, or location of an air monitor, and we leveraged this using the latitude and longitude of Pennsylvania monitoring network sites to simulate home addresses of epidemiology study subjects. The general approach of our analysis was to assess agreement between exposure quartiles based on (1) calculated WA values and (2) measured ambient pollutant concentrations for each geocoded location over a five-year period.

## 2. Materials and Methods

### 2.1. Air Quality Data

Ambient air quality data from 2011 to 2015 were compiled from the U.S. Environmental Protection Agency (EPA) Air Quality System (AQS) Data Mart [19] and the DEP [20]. From the AQS, we downloaded pre-generated data files containing hourly concentrations of PM_2.5_ (parameter code 88101), hourly and daily mean 1-h concentrations of NO_2_, SO_2_ and CO, daily 8-h running average concentrations of O_3_, and 24-h benzene concentrations for Allegheny County (2011–2013 only), measured every six days. From the DEP, we compiled daily 24-h benzene concentrations for counties other than Allegheny, also measured every six days. These specific pollutants were included in our analysis because ambient monitoring data are readily available, they are potentially related to URD activities [9,21], and have been suggested as potential mechanisms behind reported community health effects around URD operations [12,13,22,23]. Monitors located in the Philadelphia metropolitan area were excluded from all analyses, identified in the AQS data as the “Philadelphia-Camden-Wilmington, PA-NJ-DE-MD core-based statistical area”, and in the DEP data as Chester, Marcus Hook, Swarthmore, Collegeville or Evansburg counties. This was carried out since there were no nearby URD operations and pollutant concentrations were likely higher due to the presence of urban emission sources.

### 2.2. Well Data

We compiled data on all unconventional gas wells in Pennsylvania for which drilling had begun before or during the period 2011 to 2015, including all data elements required to replicate the WA metrics first described by Casey et al. [13]. Well records from the DEP were used to identify latitude, longitude and first date of drilling (“spud date”) of all unconventional gas wells, as well as the earliest spud date in cases where multiple wells were drilled on a single well pad [24]. We identified all the wells in production between 2011 and 2015 using monthly DEP Production reports [25]. The state data were supplemented by information from Drilling Info [26], a subscription-based data service with detailed information on U.S. onshore oil and gas development sites. For each well identified through DEP records, we captured data from Drilling Info including spud date, stimulation date (i.e., the first day of hydraulic fracturing), production dates, well depth and average daily gas production volumes. Drilling Info dates and production volumes took precedence in our dataset, but if they were not available, values from state databases were used. Wells without spud, stimulation and production dates in either source were excluded. Dates for each well were also subjected to various checks to determine chronological feasibility. If the dates from Drilling Info were illogical (e.g., first production date before spud date), then the state data was consulted. The wells for which state records could not resolve the data issue were excluded.

### 2.3. Well Activity Metric Calculations

**Figure 1:**
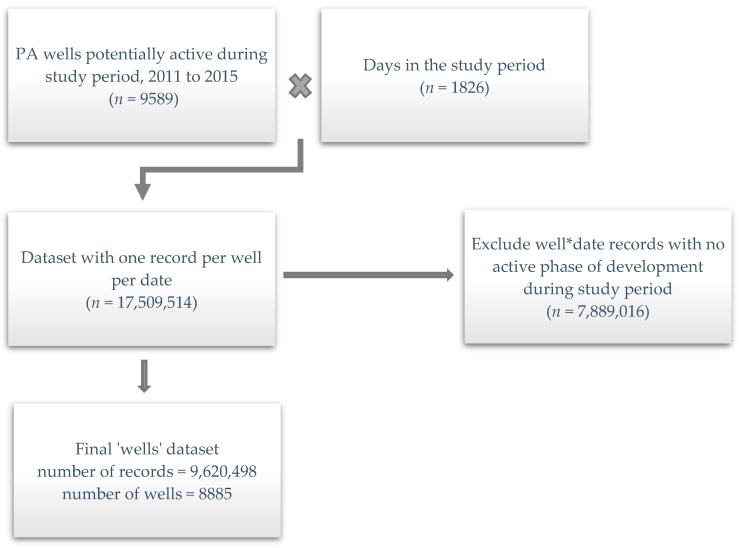
Description of process used to compile final wells dataset for analysis.

We calculated four WA metrics, one for each phase of well development, following methods first described by Casey et al. [13] and using the steps below:

Step 1—For each well, we used spud, stimulation and production dates to either assign a phase of development for every day of the study period or indicate an inactive period for the well (i.e., between two phases). Beginning and ending dates of each phase were determined as follows: (1) pad preparation began 30 days before the spud date for the first well on a pad, (2) the drilling phase began on the spud date and lasted between one and 30 days depending on well depth, (3) the fracturing phase lasted seven days, beginning with the stimulation date, and (4) the production phase began with the first production date and ended with either the last production date or the end of 2015. After assigning a phase to each combined record (i.e., one record per date, per well), we kept only those with one of the four development phases assigned, since wells did not contribute to any of the WA metric calculations on inactive dates (Figure 1).

Step 2—We joined air monitoring records to the ‘wells’ dataset described in Step 1, by date. The resulting dataset thus included one record for each monitor and well combination, for each day of the study period on which the well was active. For each record in the dataset, we calculated the distance between the monitor and the well using the SAS GEODIST function [27].

**Figure 2:**
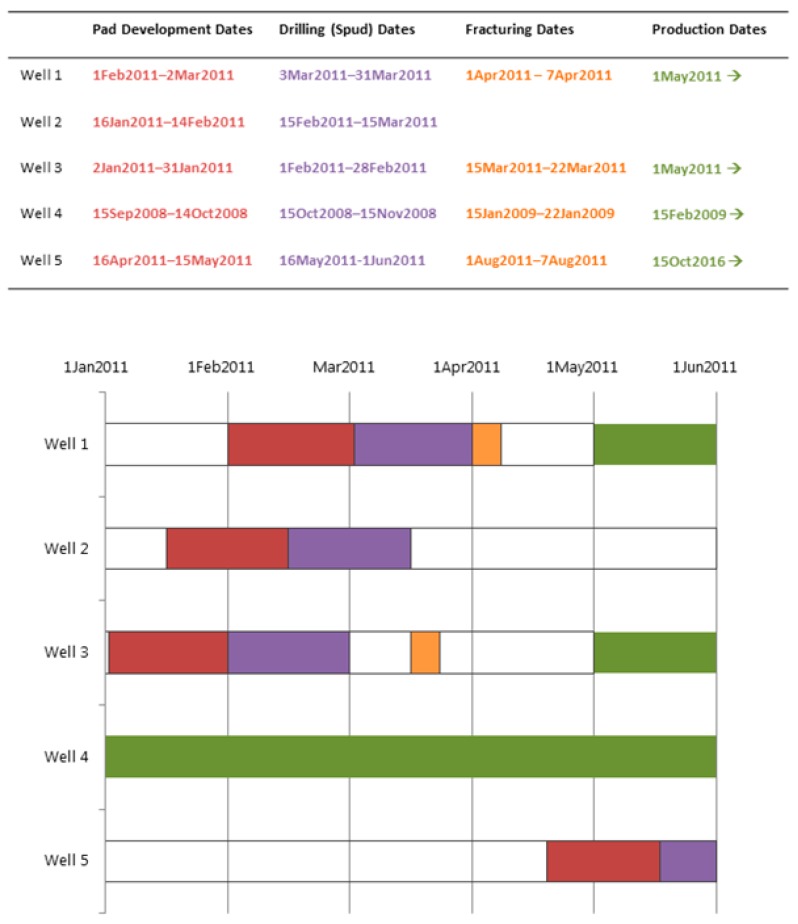
Illustration of how date ranges for phases of well development were used to calculate the four WA metrics. Note that this illustration includes only five of the 8885 wells in the study, and a portion (January to June 2011) of the 5-year study period.

**Figure 3:**
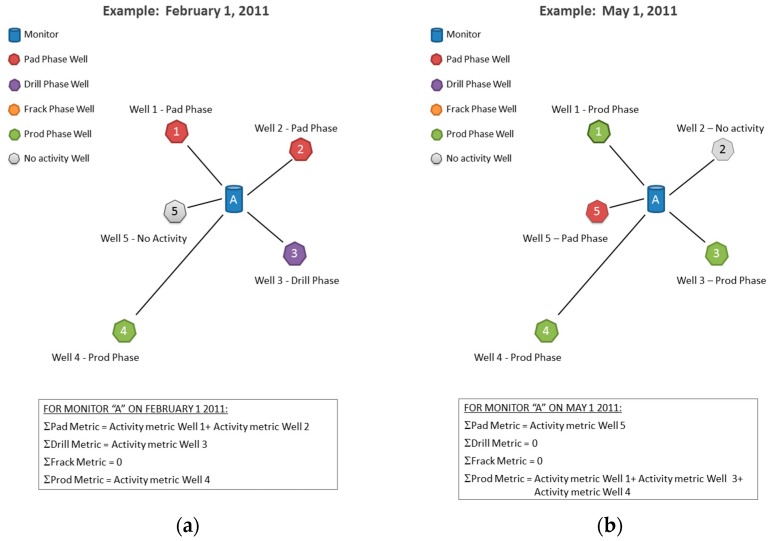
Illustration of the method used to calculate daily WA metrics on sample dates of (**a**) 1 February 2011 and (**b**) 1 May 2011 using air monitors as simulated study subject residences.

Step 3—Using data from Step 2, the four WA metrics were calculated for each monitor for every day of the study period, as shown below and described in Figure 2 and Figure 3.

Daily exposure at each monitor resulting from wells in the **pad preparation phase** was calculated as:

(1) $$\overset{n}{\underset{i=1}{\sum }}\frac{1}{d_{ij}^{2}}$$

where *n* is the number of wells in the pad preparation phase on that day and *d* is the distance (in meters) from well *i* to monitor *j*. This phase is defined as lasting 30 days prior to the spud date of the first well drilled on a well pad.

Daily exposure at each monitor resulting from wells in the **drilling phase** was calculated as

(2) $$\overset{n}{\underset{i=1}{\sum }}\frac{1}{d_{ij}^{2}}$$

where *n* is the number of wells in the drilling phase on that day and *d* is distance (in meters) from well *i* to monitor *j*. This phase is defined as lasting between 1 and 30 days beginning on the spud date, based upon percentiles of total well depth.

Daily exposure at each monitor resulting from wells in the **fracturing phase** was calculated as:

(3) $$\overset{n}{\underset{i=1}{\sum }}\frac{t_{1}}{d_{ij}^{2}}$$

where *n* is the number of wells in the fracturing phase on that day, *d* is distance (in meters) from well *i* to monitor *j*, and *t* is the total depth (in meters) of well *i*. This phase is defined as lasting 7 days beginning with the well stimulation date.

Daily exposure at each monitor resulting from wells in the **production phase** was calculated as

(4) $$\overset{n}{\underset{i=1}{\sum }}\frac{v_{1}}{d_{ij}^{2}}$$

where *n* is the number of wells in the production phase on that day, *d* is distance (in meters) from well *i* to monitor *j*, and *v* is average daily gas production volume (in cubic meters). This phase begins on the first production date and lasts through either the last production date or the end of study period.

For each activity metric, a higher value indicated a greater well density near the monitoring site, and therefore, a higher level of exposure. Activity metric calculations were repeated for each of the six pollutants, using the subset of monitors and the days for which air samples of the pollutant were available.

### 2.4. Analysis

Our final analysis file included one record for each monitor on each day, containing the mean pollutant concentration measured at the site on that day, and the four calculated WA values based on proximity of relevant wells to the latitude/longitude of the site. Continuous values of phase-specific WA across all monitors and days of the study period were divided into quartiles corresponding to ‘very low’, ‘low’, ‘medium’ and ‘high’ exposure categories. Since the calculations are based on a subset of wells across the state that were actively in the pad preparation, drilling, fracturing or production phase on that day, exposure at a particular monitoring site could be ‘low’ for one phase and ‘high’ for another phase on the same day. WA-based exposure categories also varied between monitors on any given day because of the different proximity of wells to their geocoded location. Likewise, for each of the six pollutants, we divided ambient concentrations across all the monitors and days of the study period into quartiles corresponding to ‘very low’, ‘low’, ‘medium’ and ‘high’ exposures.

Weighted kappa statistics with 95% confidence intervals were used to assess agreement between exposure categories based on pollutant concentrations and WA, interpreting the strength of agreement as follows: 0.01–0.20: none to poor; 0.21–0.40: fair; 0.41–0.60: moderate; 0.61–0.80: substantial; 0.81–1.0: almost perfect agreement [28]. We further examined 4 × 4 tables of exposure categories based on the two methods to determine the proportion of pairs in agreement, or perhaps of more interest, in extreme disagreement (e.g., high using one method/very low using the other method, and vice versa) and to assess whether values tended to fall within adjacent or non-adjacent categories in the event that the two methods disagreed.

We also conducted sensitivity analyses in which monitors with no unconventional gas wells within either a 10-km (km) or 30-km radius were excluded. Although WA metrics do not apply a buffer zone, we tested whether results would differ with closer well proximity. We also performed the analysis using 90- and 180-day rolling average pollutant concentrations in place of single-day concentrations, for consistency with what were considered relevant exposure periods in the Pennsylvania-area epidemiology studies of migraine, sinus symptoms and fatigue [14] and adverse birth outcomes [13], respectively.

## 3. Results

**Figure 4:**
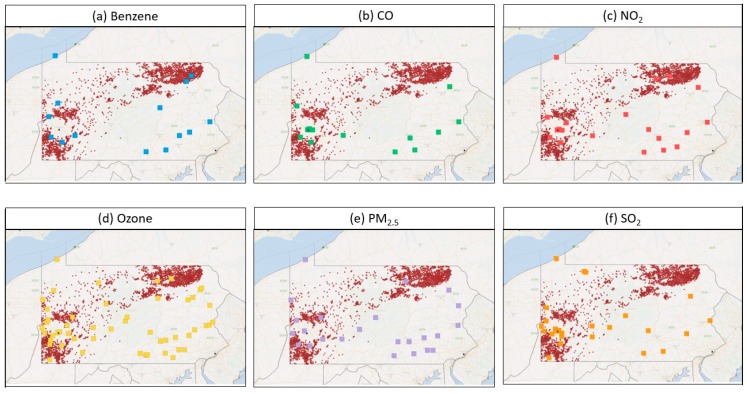
Location of unconventional gas wells (red circles) in Pennsylvania in any phase of development between 2011 and 2015, and monitoring sites (colored squares) where ambient air samples of (**a**) benzene, (**b**) CO, (**c**) NO_2_, (**d**) O_3_, (**e**) PM_2.5_ and (**f**) SO_2_ were collected, and which served as simulated study subject residences.

**Figure 5:**
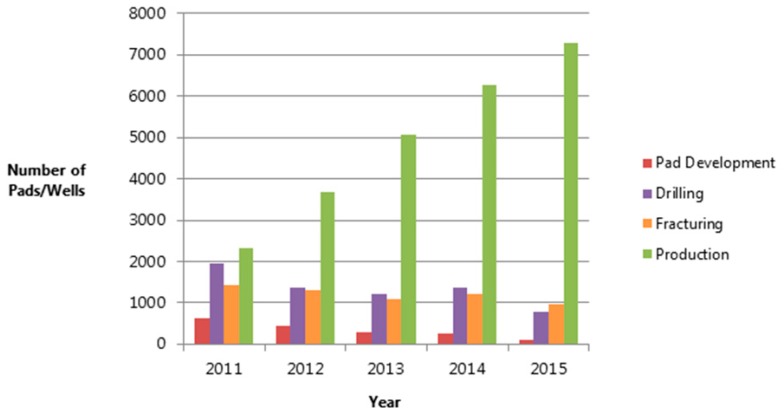
Number of unconventional gas wells in Pennsylvania by phase of development, 2011–2015.

Of the 9589 unconventional gas wells spudded before or during 2011 to 2015, we identified 8885 in any phase of development in Pennsylvania between 2011 and 2015, concentrated in the northeast and southwest portions of the state. There were 704 wells spudded prior to 2011, but since we found no evidence of further development, they were excluded from the analysis. The locations of the wells relative to monitoring sites for the six pollutants are shown in Figure 4. Well development decreased over time, however, as there were over 7000 producing wells by the end of 2015 (Figure 5). Our calculated distribution of wells by year and development phase matched that presented by Rasmussen et al. [12] for overlapping study years (2011–2012), indicating that we were able to replicate their methodology of assigning wells in the state to each of the four phases.

Across the state, ambient air samples of the six pollutants were collected at 76 monitoring sites, excluding Philadelphia-area monitors. A description of monitoring sites and quartiles of both daily mean and daily maximum pollutant concentrations is shown in Table 1. The number of monitors varied only slightly over the five-year study period but more markedly across pollutants; for example, O_3_ was measured at the most monitors across the state (*n* = 53), and CO and benzene were measured at the fewest (*n* = 16 and 15, respectively). For each WA metric, median distance between monitors and wells generally decreased with increasing exposure category, with minimum distances of 0.3–7 km in the highest exposure intensity groups (Table 2).

**Figure 6:**
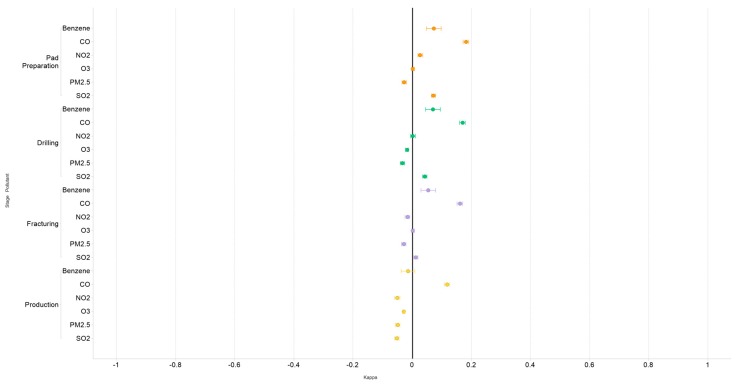
Weighted kappa statistics assessing agreement between quartiles of exposure for the four WA metrics and quartiles of daily mean pollutant concentrations, Pennsylvania unconventional gas wells, 2011–2015.

For each pollutant, we found fair to poor correlations between exposure categories derived from WA and ambient air concentrations, with almost all the weighted kappa coefficients falling between −0.1 and 0.1 and none exceeding −0.2 or 0.2 (Figure 6). Some of the coefficients were negative, indicating significant disagreement between the two methods. CO had the highest level of agreement between the two methods, but even these correlations were considered poor. In the sensitivity analyses, we saw little impact on weighted kappa statistics for any of the pollutants when including only monitors having at least one well within a 10-km or 30-km radius (Table 3). Similarly, there was no change in results when 90- or 180-day averaging periods were used in place of same-day exposure values (Figures S1 and S2).

**Figure 7:**
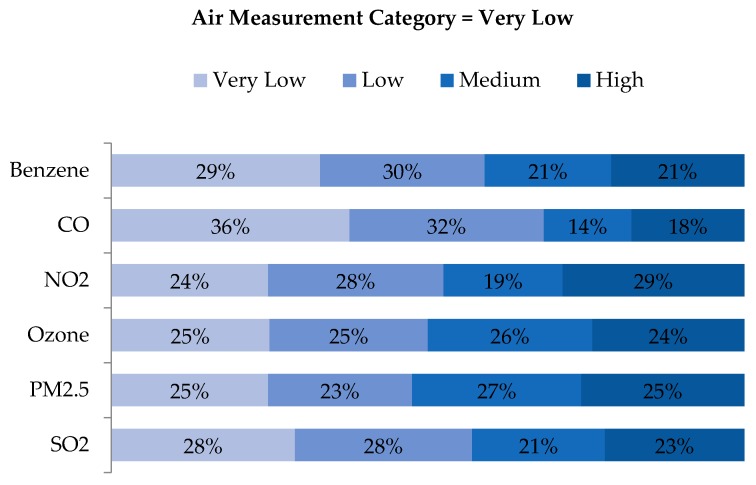
Distribution of exposure category based on WA metrics for all observations with ambient air measurements indicating ‘Very Low’ exposure, by pollutant.

**Figure 8:**
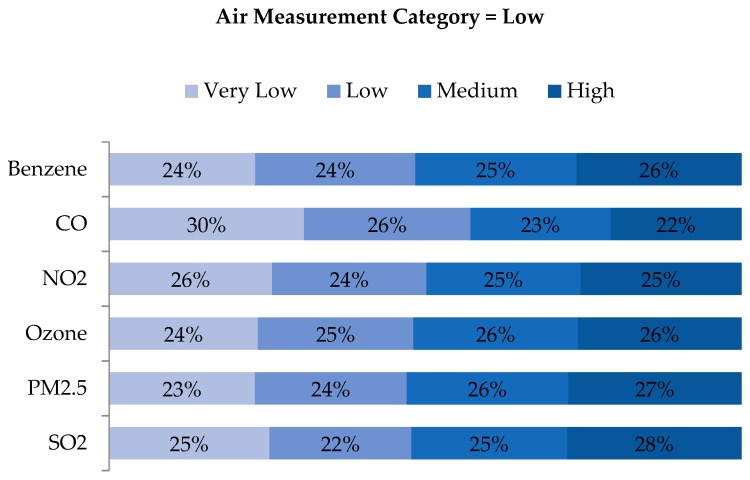
Distribution of exposure category based on WA metrics, for all observations with ambient air measurements indicating ‘Low’ exposure, by pollutant.

**Figure 9:**
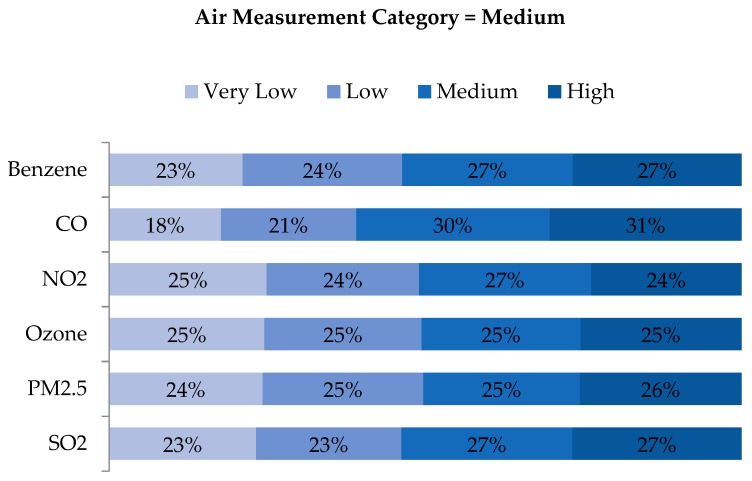
Distribution of exposure category based on WA metrics, for all observations with ambient air measurements indicating ‘Medium’ exposure, by pollutant.

**Figure 10:**
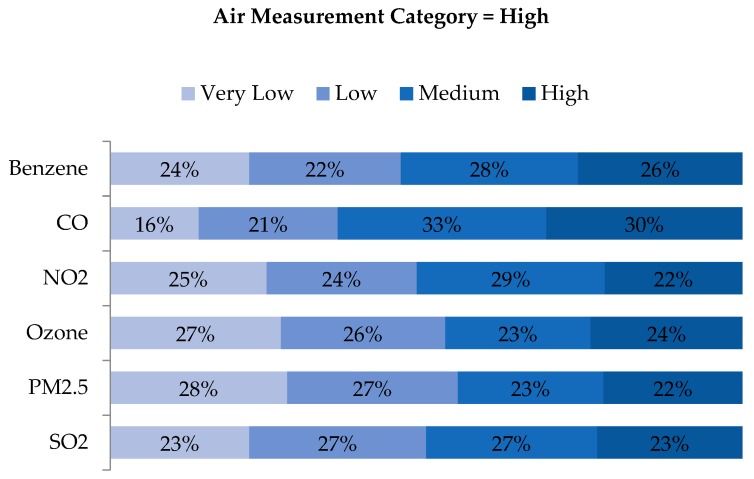
Distribution of exposure category based on WA metrics, for all observations with ambient air measurements indicating ‘High’ exposure, by pollutant.

An examination of quartile distributions for each pollutant (i.e., rows of air concentration quartiles vs. columns of WA quartiles) showed that disagreement was not limited to adjacent cells (e.g., ‘Low’ being classified as either ‘Very Low’ or ‘Medium’), but rather distributed across quartiles (Figure 7, Figure 8, Figure 9 and Figure 10). As shown in Figure 7, the locations classified as ‘Very Low’ based on air measurements were classified as ‘Very Low’ based on WA only 24%–36% of the time, depending on the pollutant. Categories for benzene and CO agreed to a greater extent than the other pollutants, but in both cases, matched just 29% and 36% of the time, respectively. Perhaps more notably, locations classified as ‘Very Low’ based on air measurements, were classified as ‘Medium’ or ‘High’ based on WA 32%–52% of the time. Conversely, locations classified as ‘High’ based on air measurements were also classified as ‘High’ based on WA only 22%–30% of the time but classified as ‘Very Low’ or ‘Low’ based on WA 37–55% of the time (Figure 10). Benzene, O_3_ and PM_2.5_ categorized as ‘High’ based on the monitors were categorized as ‘Very Low’ or ‘Low’ based on WA 46%, 53%, and 55% of the time, respectively. Examination of the ‘Low’ and ‘Medium’ categories based on air measurements yielded similar results (Figure 8 and Figure 9).

## 4. Discussion

Recent Pennsylvania-area studies using WA metrics as a proxy for URD exposure have reported statistically significant associations between URD and preterm birth [13]; asthma exacerbation [12]; and sinus symptoms, migraine, and fatigue [14]. Although the potential for measurement error and exposure misclassification was acknowledged in these studies, neither the extent to which this may be occurring nor the potential impact on resulting effect estimates have been quantitatively assessed.

In our analysis, we did not validate WA metrics using Pennsylvania air monitoring data as a gold standard, nor did we directly evaluate whether studies using WA metrics produced reliable health risk estimates. Rather, we addressed a more basic point—whether these calculated exposure estimates agreed with those from air sampling data, and therefore, could be considered suitable surrogates of exposure when air measurement and/or detailed modeling data are unavailable. The question we essentially asked was, if these monitoring sites were instead a sample of epidemiology study subjects’ homes with monitors placed outside the front door, how well does the categorization of exposure agree between the two methods? We found that they did not agree well at all with the same exposure quartile assigned in roughly one in four observations, and the opposite category assigned for roughly 25%. The implication of these results was that using this methodology in epidemiology studies can result in significant exposure misclassification and therefore, uncertainty around reported risk estimates. Further study including disease outcomes and exposure classification of cases and controls would be needed to fully understand the nature and degree of the misclassification. However, while nondifferential exposure misclassification is generally assumed to bias risk estimates toward the null, this cannot be assumed with polychotomous data, particularly in the presence of significant misclassification between nonadjacent categories as seen in nearly 50% of the observations in our analysis. Several papers on this topic have urged caution when interpreting results with this type of misclassification, even if nondifferential [29,30,31].

In epidemiology studies with point-source emissions as the exposure of interest, either proximity surrogates or dispersion modeling are often used to categorize or estimate exposure. Proximity surrogates are a crude yet simple method requiring only knowledge of the distance between emission sources and exposure receptor sites (i.e., residences) to estimate exposure. They typically impose a small buffer distance around either the emission source or the receptor in order to more reliably apportion exposure to the point-source of interest, however this limits the study population to residents with the emission source(s) within close proximity of their house. In contrast, dispersion models are considered higher quality, and can be used to predict exposure across a wide geographic area [15,16]. Studies using dispersion models do not have to constrain their study population size based on distance from emission sources and the models can be validated against measured air concentrations. However, they are more data- and labor-intensive, requiring at a minimum, emissions, background pollution and meteorological data.

To date, many URD epidemiology studies have used proximity surrogates to categorize exposure, for example, defining the population at risk as those with at least one well within a given distance of their residence, and then stratifying this “exposed” group into quantiles based on well density around the residence [22,23,32,33,34]. Other studies have built on this approach. Researchers from Colorado developed an intensity-adjusted inverse distance weighted well count metric, incorporating not only well density, but also phase of well development, production volume, and air pollutant emission rates, in order to distinguish “high intensity” events, such as hydraulic fracturing or multi-well pad development, from “low intensity” events, such as production at a single well [35]. A recent systematic review [11] concluded that existing epidemiology studies provide limited evidence of URD-related health impacts, particularly worsening of asthma and other self-reported symptoms, but less consistent evidence of adverse birth outcomes, which has been the focus of several studies [13,23,32,33,34]. While study quality is improving over time, the authors of the review conclude that evidence of URD-related health effects is still inadequate to guide policy, particularly because of the reliance on indirect measures of exposure [11].

Our analyses indicate that exposure quartiles derived from WA models at the latitude and longitude of a monitoring site, despite incorporating well characteristics such as phase of development, drilling depth, and production volumes, demonstrate poor agreement with exposure quartiles based on actual ambient monitoring data. This was true even when limiting the analysis to monitoring sites with at least one well within 10 km and 30 km, with virtually no change in weighted kappa coefficients. These results challenge some of the assumptions underlying WA methodology as surrogates for URD-related air quality impacts: that URD sites are homogeneous in terms of air emissions; that exposure at a residence is determined by well emissions without consideration of pollutant dispersion or other pollutant sources and that all wells in the state contribute to the personal exposure of all residents in the state.

There were several limitations to our analysis. First, we did not address other potential exposure pathways related to URD operations, such as water, noise, traffic or stress, although all of these could be represented by distance-based surrogates, and health effects attributed to increased well activity around a residence could potentially be explained by any of these. While not diminishing the potential importance of these pathways, we focused our analysis on the air pathway, because in our view, it was the one which WA metrics were designed to describe. Recent work that incorporated air emissions from compressor stations, impoundments and flaring events into WA metric calculations speak to the emphasis placed on the air pathway in the Marcellus epidemiology studies [36].

Secondly, our analysis was limited to only six pollutants, although additional pollutants associated with URD, particularly VOCs, have been a source of concern [37,38]. We included pollutants in the analysis that had been suggested as adversely impacting community health around URD operations, and for which ambient measurements were readily available from both state and federal sources [9,19]. Information on speciated VOCs, aside from benzene, were too sparse to include in this analysis. This is a limitation of our analysis, as VOCs and other hazardous air pollutants have been highlighted in recent quantitative risk assessments [37,38,39,40]. Recent summaries of air monitoring data in Pennsylvania [38] and Colorado [39,40] have found only sporadic exceedances of health-based comparison values, however, interpretation of the risk posed by the measured concentrations can differ depending on the comparison values used [39]. These recent assessments also identified the relative scarcity of air toxics monitoring data as a limitation.

Pennsylvania air monitors are intentionally sited in areas with high population density and/or potentially high levels of contaminants [41]. Some are in regions with dense URD activity, while others have no wells nearby. A potential criticism of our study is that depending on the location, monitors may reflect not only URD-related emissions, but also emissions from other industrial and mobile sources, making a simple comparison between monitored concentrations and WA metrics invalid. We argue that this is precisely the weakness of the WA approach. WA metrics differentiate levels of exposure based entirely on location and characteristics of unconventional wells, and do not attempt to adjust for emissions from other sources of exposure. A study subject may live next to a major highway, an industrial site, or a mining area, yet his or her exposure estimate considers none of those. Information provided by Rasmussen [12], (see Figure 3) and Casey [13], see (Figure 2) indicate that while some study subjects lived in close proximity to well sites, most lived in counties far removed from drilling activity, similar to the geographic distribution of monitoring sites available for our analysis. Although cases and controls were grouped into relative exposure categories in these studies, the potential for demographic or environmental factors other than well activity to explain geographic differences in risk are clear. Furthermore, our sensitivity analysis confirmed that even if WA calculations are limited to subjects in areas with nearby URD activity, the performance of the metrics did not improve.

A recent paper by Koehler et al. [36] compared the risk of asthma using four different proximity-based exposure methods: WA (as used in Rasmussen et al. [12], Casey et al. [13] and Tustin et al. [14]); WA + inclusion of compressor station locations (first described in Koehler et al. [36]); distance to nearest drilled well (as used in Rabinowitz et al. [42]); and inverse distance weighting of drilling-phase wells within 10 km of residence (as used in McKenzie et al. [23] and Stacy et al. [32]). The range of risk estimates for highest vs. lowest exposure category ranged from 1.19 (95% confidence interval: 1.01–1.41) to 4.43 (95% CI: 3.75–5.22). This is significant variability, with WA metrics producing the highest estimated asthma risk. This demonstrates the instability of risk estimates in epidemiology studies with distance-based exposure metrics and the need for study methods that minimize the likelihood of measurement error.

We hypothesized that exposure intensity based on WA metrics, which do not incorporate emissions from URD sites or other point or mobile sources, air monitoring data, or pollutant dispersion modeling, would show little agreement with exposure intensity based on actual air measurements. Our results confirm this hypothesis. WA metrics allow exposure and health risks to be estimated for each phase of well development, an important consideration when characterizing air emissions and potential community exposures near well sites. However, our results suggest that they do not accurately distinguish levels of exposure to pollutants via the air pathway and do not follow a predictable trend when exposure classifications do not agree. We underscore this uncertainty and urge a more cautious interpretation when using these studies in community engagement or policy decisions. Epidemiology studies employing direct measures of exposure or dispersion modelling with robust inputs that can distinguish and estimate URD-related exposure over a wide geographic area are needed in order to assess potential health impacts related to these operations.

## 5. Conclusions

Our study evaluated agreement between exposure categories based on ambient air measurements and estimates of WA. When comparing very low vs. high categories, we found that the two methods agreed ~25% of the time and produced opposite classifications ~25% of the time. Our analysis suggests that these WA metrics do not adequately distinguish categories of air pollutant exposure and employing them in epidemiology studies can result in significant misclassification of exposure.

## Figures and Tables

**Table 1:** Number of monitoring sites and quartiles of daily mean and maximum concentrations by pollutant, all Pennsylvania monitoring sites except Philadelphia-metropolitan area, in 2011–2015. The quartiles of mean concentrations were used as cut-points to define very low, low, medium and high exposure categories based on air monitoring data.

			Daily Mean *			Daily Maximum		
Pollutant	Number of Monitors	Number of Observations **	25th Percentile	50th Percentile	75th Percentile	25th Percentile	50th Percentile	75th Percentile
Benzene (ppbv)	15	3410	0.11	0.16	0.23	--	--	--
CO (ppm)	16	21,616	0.03	0.18	0.31	0.1	0.3	0.6
NO_2_ (ppb)	21	28,777	3.5	6.5	10.6	8.0	15.0	24.0
O_3_ (ppb)	53	80,620	20.7	27.8	34.8	30.0	38.0	47.0
PM_2.5_ (µg/m^3^)	24	37,378	6.5	9.8	14.0	13.1	18.8	25.5
SO_2_ (ppb)	25	38,449	0.3	1.3	2.9	1.5	4.0	8.0

* Daily 8-h running average concentrations for ozone; daily 1-h mean and maximum (except benzene) for all other pollutants. ** Reflects both the number of monitors measuring the pollutant, and the number of days samples were taken at each over the 5-year period.

**Table 2:** Median distances between monitoring sites and statewide wells by phase of well development and exposure category (excluding Philadelphia-area monitors), 2011–2015 (minimum and maximum distances in italics).

**Pollutant**	**Pad Preparation Metric**				**Drilling Metric**			
	**Very Low**	**Low**	**Medium**	**High**	**Very Low**	**Low**	**Medium**	**High**
Benzene	264 km *(34; 448)*	230 km *(21; 450)*	184 km *(11; 449)*	168 km *(1; 439)*	257 km *(39; 450)*	217 km *(29; 450)*	196 km *(12; 445)*	188 km *(1; 439)*
CO	245 km *(37; 447)*	191 km *(18; 450)*	215 km *(9; 454)*	129 km *(7; 454)*	244 km *(36; 450)*	195 km *(17; 454)*	240 km *(10; 454)*	127 km *(4; 449)*
NO_2_	253 km *(22; 448)*	205 km *(134; 352)*	184 km *(11; 454)*	131 km *(1; 454)*	249 km *(36; 450)*	195 km *(18; 450)*	194 km *(11; 454)*	133 km *(1; 449)*
O_3_	258 km *(22; 460)*	197 km *(16; 464)*	178 km *(9; 464)*	136 km *(0.6; 460)*	248 km *(31; 464)*	189 km *(18; 464)*	183 km *(11; 459)*	136 km *(0.6; 460)*
PM_2.5_	259 km *(32; 460)*	213 km *(19; 461)*	175 km *(14; 464)*	147 km *(1; 454)*	253 km *(36; 460)*	201 km *(26; 464)*	174 km *(12; 464)*	142 km *(1; 449)*
SO_2_	235 km *(33; 460)*	181 km *(18; 460)*	178 km *(9; 459)*	126 km *(2; 460)*	232 km *(31; 460)*	186 km *(17; 459)*	192 km *(9; 459)*	119 km *(2; 460)*
	**Fracturing Metric**				**Production Metric**			
	**Very Low**	**Low**	**Medium**	**High**	**Very Low**	**Low**	**Medium**	**High**
Benzene	251 km *(56; 448)*	220 km *(27; 450)*	222 km *(15; 445)*	146 km *(1; 444)*	219 km *(2; 441)*	212 km *(3; 450)*	213 km *(5; 450)*	221 km *(0.3; 444)*
CO	242 km *(12; 450)*	212 km *(25; 454)*	246 km *(13; 454)*	144 km *(2; 454)*	219 km *(11; 441)*	205 km *(11; 450)*	232 km *(5; 450)*	247 km *(0.8; 454)*
NO_2_	246 km *(27; 450)*	203 km *(25; 454)*	197 km *(14; 454)*	129 km *(0.3;454)*	212 km *(11; 441)*	204 km *(6; 450)*	201 km *(5; 450)*	218 km *(0.3; 454)*
O_3_	247 km *(12; 464)*	195 km *(13; 464)*	184 km *(6; 464)*	140 km *(0.3; 460)*	211 km *(5; 458)*	196 km *(0.9; 461)*	192 km *(0.9; 464)*	218 km *(0.3; 460)*
PM_2.5_	250 km *(27; 464)*	205 km *(25; 464)*	180 km *(7; 464)*	165 km *(1; 454)*	211 km *(7; 455)*	194 km *(0.9; 460)*	183 km *(0.9; 464)*	221 km *(0.6; 464)*
SO_2_	231 km *(25; 460)*	191 km *(7; 460)*	212 km *(7; 459)*	132 km *(2; 460)*	200 km *(6; 458)*	199 km *(5; 460)*	237 km *(2; 460)*	256 km *(2; 460)*

**Table 3:** Results of sensitivity analysis comparing weighted kappa values for monitors with 10- and 30-km buffer zones applied, and results for all monitors regardless of distance to nearest well.

Pollutant	<10 km			<30 km			All		
	Kappa	Lower 95% CI	Upper 95% CI	Kappa	Lower 95% CI	Upper 95% CI	Kappa	Lower 95% CI	Upper 95% CI
Pad Preparation									
Benzene	0.1420	0.0326	0.2515	−0.0631	−0.1063	−0.0200	0.0729	0.0484	0.0975
CO	0.2495	0.1727	0.3263	0.0002	−0.0184	0.0180	0.1816	0.1721	0.1911
NO_2_	0.0230	−0.0233	0.0692	−0.1448	−0.1589	−0.1306	0.0268	0.0185	0.0352
O_3_	0.0122	−0.0090	0.0333	0.0258	0.0168	0.0349	0.0019	−0.0031	0.0069
PM_2.5_	0.0460	0.0149	0.0771	0.0338	0.0195	0.0480	−0.0272	−0.0345	−0.0199
SO_2_	−0.0355	−0.0750	−0.0041	−0.0417	−0.0539	−0.0295	0.0708	0.0637	0.0780
Drilling									
Benzene	−0.0532	−0.1433	0.0369	−0.0746	−0.1187	−0.0305	0.0700	0.0456	0.0943
CO	−0.0187	−0.0919	0.0544	−0.0769	−0.0940	−0.0598	0.1697	0.1603	0.1792
NO_2_	−0.1015	−0.1405	−0.0625	−0.1541	−0.1681	−0.1401	0.0020	−0.0062	0.0102
O_3_	0.0257	0.0032	0.0482	0.0270	0.0178	0.0362	−0.0183	−0.0233	−0.0134
PM_2.5_	−0.0232	−0.0572	0.0108	0.0245	0.0100	0.0391	−0.0331	−0.0404	−0.0259
SO_2_	−0.0702	−0.1038	−0.0365	−0.0506	−0.0630	−0.0383	0.0419	0.0348	0.0491
Fracturing									
Benzene	-0.0175	−0.1375	0.1025	−0.1022	−0.1488	−0.0555	0.0535	0.0290	0.0779
CO	0.0762	-0.0124	0.1648	−0.1074	−0.1277	−0.0872	0.1610	0.1515	0.1705
NO_2_	−0.0449	−0.1014	0.0116	−0.1657	−0.1814	−0.1501	−0.0166	−0.0249	−0.0084
O_3_	0.0187	−0.0112	0.0485	0.0359	0.0253	0.0465	0.0017	−0.0032	0.0067
PM_2.5_	−0.0546	−0.0958	−0.0134	0.0087	−0.0077	0.0251	−0.0296	−0.0369	−0.0224
SO_2_	−0.0843	−0.1336	−0.0349	−0.0670	−0.0817	−0.0523	0.0112	0.0041	0.0183
Production									
Benzene	−0.0554	−0.1005	−0.0104	−0.1102	−0.1446	−0.0759	-0.0141	−0.0374	0.0091
CO	−0.1281	−0.1511	−0.1050	0.0595	0.0469	0.0720	0.1172	0.1077	0.1267
NO_2_	−0.3324	−0.3446	−0.3203	−0.1656	−0.1760	−0.1553	−0.0509	−0.0590	−0.0428
O_3_	0.0187	−0.0112	0.0485	0.0123	0.0057	0.0188	−0.029	−0.0344	−0.0245
PM_2.5_	0.0454	0.0318	0.0590	−0.0124	−0.0228	−0.0021	−0.0488	−0.0561	−0.0416
SO_2_	−0.1842	−0.1969	−0.1715	−0.0927	−0.1011	−0.0842	−0.0525	−0.0596	−0.0455

## Supplementary Materials

The following are available online at https://www.mdpi.com/1660-4601/16/17/3055/s1, Figure S1: Weighted kappa statistics assessing agreement between quartiles of exposure for the four WA metrics and quartiles of 90-day average mean pollutant concentrations, Pennsylvania unconventional gas wells, 2011–2015, Figure S2: Weighted kappa statistics assessing agreement between quartiles of exposure for the four WA metrics and quartiles of 180-day average mean pollutant concentrations, Pennsylvania unconventional gas wells, 2011–2015.
