# Supplementary material for: Assessing Agreement in Exposure Classification between Proximity-Based Metrics and Air Monitoring Data in Epidemiology Studies of Unconventional Resource Development
Source: Int J Environ Res Public Health. 2019 Aug 23;16(17):3055. doi: 10.3390/ijerph16173055 (PMC6747456; doi:10.3390/ijerph16173055)
Supplement: Supplementary file 1 [file ijerph-16-03055-s001.pdf]

Supplementary Materials: The following are available online at www.mdpi.com/xxx/s1, Figure S1: Weighted kappa statistics assessing agreement between quartiles of exposure for the four WA metrics and quartiles of 90-day average mean pollutant concentrations, Pennsylvania unconventional gas wells, 2011-2015, Figure S2: Weighted kappa statistics assessing agreement between quartiles of exposure for the four WA metrics and quartiles of 180-day average mean pollutant concentrations, Pennsylvania unconventional gas wells, 2011-2015.

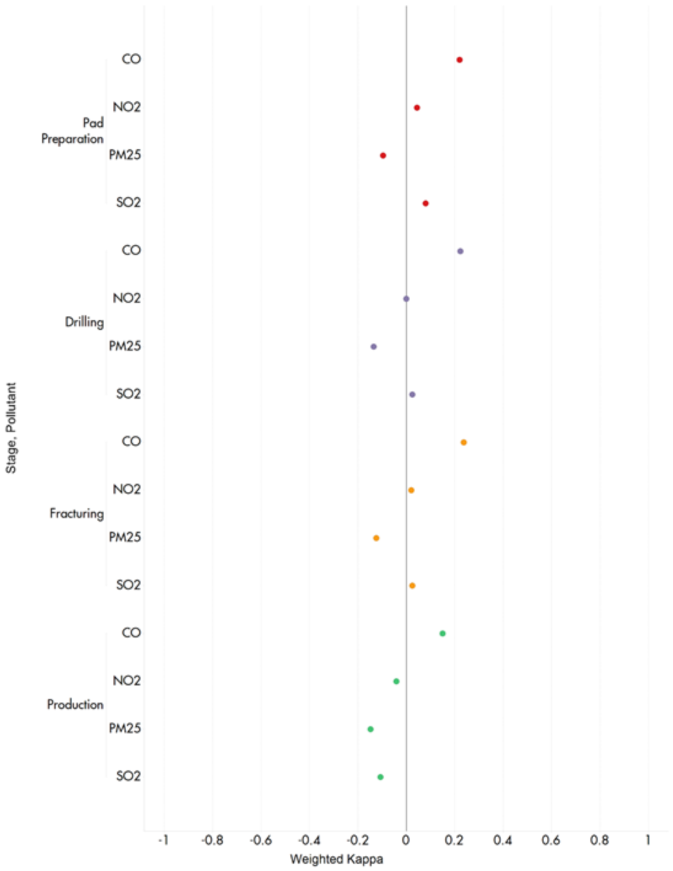

Figure S1. Weighted kappa statistics assessing agreement between quartiles of exposure for the four well activity metrics and quartiles of 90-day average mean pollutant concentrations, Pennsylvania unconventional gas wells, 2011–2015.

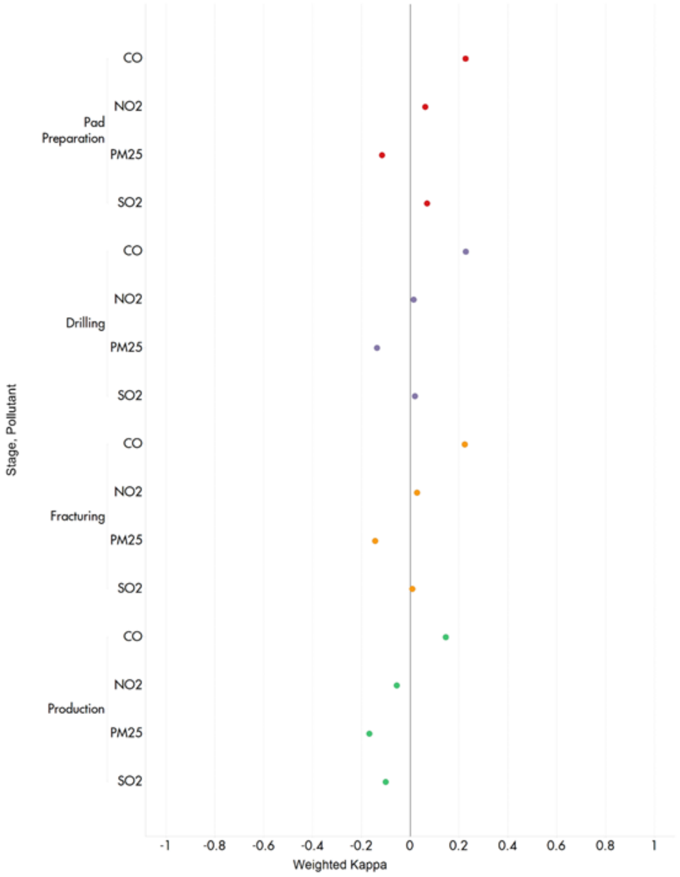

Figure S2. Weighted kappa statistics assessing agreement between quartiles of exposure for the four well activity metrics and quartiles of 180-day average mean pollutant concentrations, Pennsylvania unconventional gas wells, 2011–2015.
